# Enteral feeding timing and clinical outcomes in neonates with hypoxic-ischemic encephalopathy undergoing therapeutic hypothermia: a retrospective cohort study highlighting the challenge of confounding by indication

**DOI:** 10.3389/fped.2026.1818709

**Published:** 2026-04-23

**Authors:** Weihong Yue, Gege Liu, Lu Lin, Xia Liu, Ya Hu

**Affiliations:** 1 Department of Neonatology, Children’s Hospital of Chongqing Medical University, Chongqing, China; 2 National Clinical Research Center for Children and Adolescents’ Health and Diseases, Chongqing, China; 3 Ministry of Education Key Laboratory of Child Development and Disorders, Chongqing, China; 4 China International Science and Technology Cooperation Base of Child Development and Critical Disorders, Chongqing, China; 5 Chongqing Key Laboratory of Structural Birth Defect and Reconstruction, Chongqing, China

**Keywords:** enteral nutrition, hypoxic-ischemic encephalopathy, indication confounding, neonates, therapeutic hypothermia

## Abstract

**Objective:**

To analyze real-world feeding decisions in neonates with hypoxic-ischemic encephalopathy (HIE) undergoing therapeutic hypothermia (TH), assess potential confounding by indication, and clarify how this bias may affect the interpretation of the association between feeding timing and clinical outcomes.

**Methods:**

We conducted a single-center retrospective cohort study involving 94 consecutive neonates with moderate to severe HIE who received standardized TH at the Children's Hospital of Chongqing Medical University from March 2024 to March 2025. These neonates were assigned to either the early enteral feeding (EEF) group (initiated during TH/rewarming, *n* = 48) or the delayed enteral feeding (DEF) group (initiated post-TH/rewarming, *n* = 46). The primary outcome was gastrointestinal adverse events (GIAEs). We used multivariate logistic regression to adjust for confounders and performed subgroup analyses to explore feeding safety across different disease severity levels.

**Results:**

The DEF group had significantly higher rates of invasive ventilation (28.3% vs. 6.3%), hemodynamic support (82.6% vs. 29.2%), and peripherally inserted central catheter (PICC) placement (50.0% vs. 14.6%) (all *P* < 0.001), suggesting confounding by indication. No significant differences existed between the two groups in GIAEs (22.9% vs. 21.7%, *P* = 0.891), feeding intolerance (18.8% vs. 17.4%, *P* = 0.864), or necrotizing enterocolitis (NEC) (4.2% vs. 4.3%, *P* > 0.999). After adjustment, EEF was not independently associated with GIAEs (OR = 0.75, 95% CI: 0.23–2.44, *P* = 0.636), but it shortened time to feeding initiation and parenteral nutrition duration, and reduced hospitalization costs (all *P* < 0.05). In the severe HIE subgroup (*n* = 11), NEC incidence was 18.2%, higher than the overall cohort (4.3%, *P* = 0.042), though no intergroup difference was seen due to the small sample size.

**Conclusions:**

Confounding by indication may have contributed to the observed associations between early feeding and outcomes in prior studies. In clinically stable neonates with moderate HIE, early enteral feeding (≤20 mL/kg/day, with gradual advancement <20 mL/kg/day) was associated with shorter parenteral nutrition duration and reduced hospitalization costs. However, these findings should be interpreted with caution given the potential for residual confounding, and future prospective studies are needed for confirmation.

## Introduction

Hypoxic-ischemic encephalopathy (HIE) remains a leading cause of neonatal mortality and long-term neurodevelopmental disability, with an estimated 100,000 affected infants annually in China alone (1). Therapeutic hypothermia (TH) is currently the only evidence-based neuroprotective intervention and is the standard of care for moderate to severe HIE (2). However, the optimal timing for initiating enteral feeding in infants undergoing TH is still debated in neonatal intensive care units. On one hand, both perinatal asphyxia and TH may theoretically compromise intestinal perfusion and motility, raising concerns about the safety of early feeding. On the other hand, prolonged parenteral nutrition due to delayed enteral feeding is associated with increased risks of infection, mucosal atrophy, and other complications (3, 4).

While several retrospective studies and small randomized trials have suggested that early enteral feeding is safe and feasible, a critical methodological flaw limits the interpretability of this evidence (3–12): inadequate control for indication bias. In clinical practice, the decision of when to initiate feeding is often highly correlated with the infant's baseline severity of illness. Sick infants are more likely to have feeds withheld or delayed, making it difficult to determine whether observed differences in outcomes are attributable to the feeding strategy itself or to the underlying disease severity. This form of confounding, if not properly addressed, may lead to an overestimation of the benefits of early feeding and potentially compromise the safety of care in more vulnerable neonates (13).

More specifically, existing studies in this area are limited in three important ways. First, most have adjusted only for basic demographic characteristics and have not accounted for key indicators of illness severity, such as the need for invasive ventilation or vasoactive support, thereby leaving substantial residual confounding (3, 5, 6, 9). Second, few have performed stratified analyses based on disease severity, which limits the applicability of findings to specific subgroups (3, 5, 7, 9, 12). Third, there has been little effort to quantify how real-world feeding decisions are individualized, which restricts the external validity of the current evidence (3–10). As a result, causal inferences drawn from observational studies on this topic remain highly susceptible to bias.

The present study does not aim to offer definitive clinical recommendations. Rather, its purpose is to methodologically examine the extent to which indication confounding influences the observed relationship between enteral feeding timing and outcomes in neonates with HIE treated with TH. Specifically, we seek to: (1) characterize differences in clinical severity between infants receiving early vs. delayed enteral feeding; (2) compare unadjusted and multivariable-adjusted estimates to illustrate how confounding affects the apparent association between feeding timing and clinical outcomes; and (3) highlight the inherent limitations of small observational studies in supporting causal conclusions. Through this approach, we aim to provide methodological insights for researchers interpreting real-world data and underscore the importance of rigorous design in future studies of ental feeding during therapeutic hypothermia.

## Materials and methods

### Definitions

All definitions in this study were formulated in accordance with internationally and nationally recognized clinical guidelines and high-impact previous studies, with detailed specifications to ensure the reproducibility of study results:

(1) Early enteral feeding (EEF): Enteral feeding initiated during therapeutic hypothermia (TH) (rectal temperature maintained at 33.5 ℃–34.5 ℃ for 72 consecutive hours) or the rewarming phase (rectal temperature rewarmed to 36.5 ℃–37.5 ℃ at a rate of 0.5 ℃ per hour) (2). (2) Delayed enteral feeding (DEF): Enteral feeding initiated after the completion of TH and rewarming (rectal temperature stabilized at 36.5 ℃–37.5 ℃ for at least 1 h). (3) Gastrointestinal adverse events (GIAEs): The occurrence of feeding intolerance or necrotizing enterocolitis (NEC) of stage Ⅱ or higher, with NEC diagnosed according to the modified Bell criteria (14). (4) Feeding intolerance (FI) in clinical practice is typically identified by the manifestation of one or more of the following criteria: (1) gastric residual volume exceeding 50% of the prior feeding amount; (2) vomiting of gastric contents occurring more than twice within a 24-hour period; (3) frequent loose stools, specifically diarrhea exceeding six episodes per day; (4) abdominal distension, characterized by an increase in abdominal circumference greater than 2 cm from the baseline measurement; (5) hematochezia, indicated by the presence of visible rectal bleeding; or (6) failure to adhere to the planned enteral feeding regimen, which encompasses reduced, postponed, or interrupted feedings. Collectively, these manifestations indicate an inability to tolerate enteral nutrition (15). (5) Minimal enteral feeding: Enteral feeding dosage ≤20 mL/(kg·day) (5). (6) Slow feeding advancement: Daily increase in enteral feeding dosage <20 mL/(kg·day) (16). (7) Hemodynamic support: Continuous intravenous infusion of vasoactive agents [e.g., dopamine ≥5 μg/(kg·min), norepinephrine ≥0.1 μg/(kg·min)] for a duration of at least 2 h (17). In our unit, dopamine is frequently initiated to manage bradycardia during therapeutic hypothermia and to support microcirculation in infants with perinatal asphyxia, which may contribute to the high rate observed. (8) Full enteral feeding: Achievement of an enteral milk intake of 120 mL/(kg·day) with discontinuation of parenteral nutrition; intravenous clear fluids could be administered as needed at a dosage ≤10 mL/(kg·day) (18, 19). (9) Severe neurological injury: Electroencephalogram (EEG) showing burst suppression/isoelectricity (9), magnetic resonance imaging (MRI) demonstrating basal ganglia/thalamic lesions or restricted diffusion in ≥2 cerebral lobes, or intraventricular hemorrhage of grade Ⅲ or higher (8). (10) Late-onset sepsis: Positive blood culture results obtained after ≥3 days of parenteral nutrition administration (20). (11) Neonatal hypoglycemia: Blood glucose level <2.2 mmol/L within 48 h after birth and <2.5 mmol/L after 48 h of birth (21). (12) Moderate-to-severe hypoxic-ischemic encephalopathy (HIE) was diagnosed according to Chinese Medical Association guidelines (22), requiring: (1) evidence of fetal distress (abnormal cardiotocography, meconium-stained amniotic fluid, or fetal scalp pH <7.1 or lactate >4.8 mmol/L); (2) postnatal compromise (5-min Apgar <6, umbilical/initial pH ≤7.00 or base deficit ≥16 mEq/L, resuscitation >3 min); and (3) neurological signs within 6 h of birth (modified Sarnat stage ≥2) (23).

### Eligibility for therapeutic hypothermia

Decisions to initiate therapeutic hypothermia were guided by national guidelines but adapted to our institutional setting. Our hospital is a freestanding children's facility without an obstetrics department; all infants were outborn and referred from peripheral delivery hospitals. Under these circumstances, umbilical cord blood gas data were available for only a subset of infants, and Apgar scores recorded in referral records may reflect reporting bias (tending toward higher values) due to medicolegal considerations.

In routine practice, we therefore applied a slightly broader acid-base threshold (base deficit ≤12 mEq/L) as one criterion for initiating hypothermia, combined with clinical evidence of moderate-to-severe encephalopathy (Sarnat stage ≥2, seizures, or abnormal aEEG). This approach acknowledges that acidosis severity exists on a continuum and that some infants with less severe metabolic disturbance may still benefit from neuroprotection when encephalopathy is present. Importantly, all infants who received hypothermia had already met the diagnostic criteria for HIE described above.

### Study design and participants

This retrospective cohort study was conducted at the same institution. Consecutive neonates admitted to the neonatal intensive care unit (NICU) between March 2024 and March 2025 were included if they met the following criteria: (1) admission within 6 h of birth; (2) gestational age ≥35 weeks and birth weight ≥2,000 g; (3) diagnosis of moderate-to-severe HIE as defined above; and (4) receipt of standardized therapeutic hypothermia.

Exclusion criteria were: (1) deviation from the departmental feeding protocol; (2) metabolic or genetic diseases, or congenital gastrointestinal malformations; (3) hospital stay <7 days (including withdrawal of care or early death); and (4) suspected or confirmed necrotizing enterocolitis before enteral feeding initiation. We acknowledge that exclusion of infants with hospital stay <7 days introduces survivor bias, as discussed in the limitations section. Due to incomplete data capture for some transferred-out cases, a formal sensitivity analysis including these excluded infants could not be performed.

### Nutritional and clinical management protocol

Both groups received the standardized clinical management protocol of the NICU in our hospital (Supplementary Material), and no clinical decisions were intervened in this study to ensure that the results were consistent with real-world clinical practice. The feeding management criteria described below reflect the standardized feeding protocol of our NICU during the study period and were systematically documented in the medical records of all enrolled patients.

(1) Enteral nutrition: Human milk was the first choice, and preterm formula was used if human milk was unavailable. Feeding was administered orally or via nasogastric tube with an initial dosage of ≤20 mL/(kg·day). Abdominal circumference, gastric residual volume, and bowel sounds were jointly assessed by medical staff daily. Gradual volume advancement at a rate of <20 mL/(kg·day) was performed if feeding was well tolerated. Feeding was immediately suspended for 6–8 h for re-evaluation if abdominal circumference increased by more than 2 cm or bowel sounds were less than 4 times per minute (24). (2) Parenteral nutrition: Initiated within 24–48 h after birth, the protocol was in accordance with the guidelines of the European Society for Clinical Nutrition and Metabolism (ESPEN), The parenteral nutrition formulations contained lipid emulsions, amino acids, vitamins, and trace elements (25). (3) Other clinical management: Interventions including respiratory support, anti-infective therapy, and vasoactive agent administration were individualized and implemented by the attending medical team based on the neonate's specific clinical condition. (4) Antibiotic administration: Prophylactic antibiotics were routinely initiated in most HIE infants undergoing therapeutic hypothermia, considering: ① the high risk of infection associated with invasive monitoring, endotracheal intubation, and central venous access; and ② the difficulty of excluding early-onset infection during therapeutic hypothermia, when clinical signs may be masked. Antibiotic choice and duration were determined by the attending physician based on clinical indication and culture results. (5) Human milk feeding: Human milk was the preferred choice. However, due to the outborn nature of the cohort—all infants were referred from peripheral hospitals to our children's hospital without obstetric services—mother's own milk was often not available at the time of feeding initiation, as it required transport to the ward by family members. To avoid delaying enteral feeding, standard formula was used for initial feeds when human milk was not immediately accessible.

### Outcome assessment

Primary outcome: Incidence of gastrointestinal adverse events (GIAEs) during hospitalization, including feeding intolerance and necrotizing enterocolitis (NEC) of stageⅡor higher.

Secondary outcomes: Incidences of late-onset sepsis, neonatal hypoglycemia, and severe neurological injury; time to achieve full enteral feeding, duration of parenteral nutrition, total hospital stay, and total hospitalization costs; weight gain rate and discharge weight.

Outcome adjudication: Study outcomes were independently assessed by two trained evaluators who were not involved in the clinical care or feeding decisions of the enrolled neonates. Both assessors underwent standardized training prior to data extraction and chart review. The training covered the definition of feeding intolerance, Bell staging for NEC, neuroimaging scoring methods, and criteria for other secondary outcomes. Disagreements between the two assessors were resolved through consensus discussion. It is important to note that while the assessors were not blinded to group allocation due to the nature of the intervention, we sought to minimize potential information bias through rigorous standardized training and predefined outcome criteria.

### Statistical analysis

All statistical analyses were performed using IBM SPSS Statistics version 23.0 (IBM Corp., Armonk, NY, USA). A two-tailed significance level of 0.05 was used throughout. Given the single-center retrospective exploratory nature of this study, no *a priori* sample size calculation was performed. A *post-hoc* power analysis using G*Power 3.1.9.7 showed that the statistical power for the primary outcome (incidence of GIAEs) was 78.2%. Normality of continuous variables was assessed using the Shapiro–Wilk test. Normally distributed variables are presented as mean ± standard deviation (*x¯* ± *s*), while non-normally distributed variables are presented as median [interquartile range, *M* (IQR)]. Categorical variables are reported as numbers and percentages [*n* (%)]. For between-group comparisons, independent samples *t*-tests (with Levene's test for homogeneity of variance) were used for normally distributed continuous variables, and Mann–Whitney *U* tests for non-normally distributed continuous variables. Categorical variables were compared using the chi-square test or Fisher's exact test when expected frequencies were insufficient. For confounder adjustment, we acknowledge that this approach does not fully eliminate confounding by indication, particularly given the marked baseline imbalances between groups. More robust causal inference methods (e.g., propensity score matching, inverse probability of treatment weighting) were not applied due to the exploratory nature of this study and the limited sample size, which would have further compromised statistical power. Therefore, our findings should be interpreted as associations rather than causal effects. Given the limited number of outcome events (GIAEs: *n* = 21) and the exploratory nature of this study, we retained four key clinically relevant confounders in the multivariable model: HIE severity (moderate vs. severe), need for invasive mechanical ventilation, use of vasoactive agents, and early feeding status. These variables were selected based on strong clinical rationale and prior literature, as they are well-established predictors of both feeding decisions and clinical outcomes in this population. Early feeding status is the primary exposure of interest and was therefore retained despite sample size limitations. The events-per-variable ratio was approximately 5, which is below the conventional threshold for confirmatory analyses. However, this ratio is considered acceptable for exploratory studies where the goal is hypothesis generation rather than definitive causal inference. Nevertheless, the wide confidence intervals reflect limited statistical power, and findings should be interpreted as hypothesis-generating. Variables were forced into the model regardless of their *P*-values in univariate analyses, as these factors are well-established predictors of both feeding decisions and clinical outcomes. Collinearity was assessed using the variance inflation factor (VIF), and all VIF values were <10, indicating no significant collinearity. Model fit was evaluated using the Hosmer-Lemeshow goodness-of-fit test. Subgroup analyses were performed stratified by HIE severity (moderate/severe), ventilation mode (invasive ventilation/non-invasive ventilation/no ventilation support), and hemodynamic status (support required/no support required). Health economics analysis involved comparison of total hospitalization costs between the two groups.

## Results

### Baseline characteristics of the study subjects

**Figure 1:**
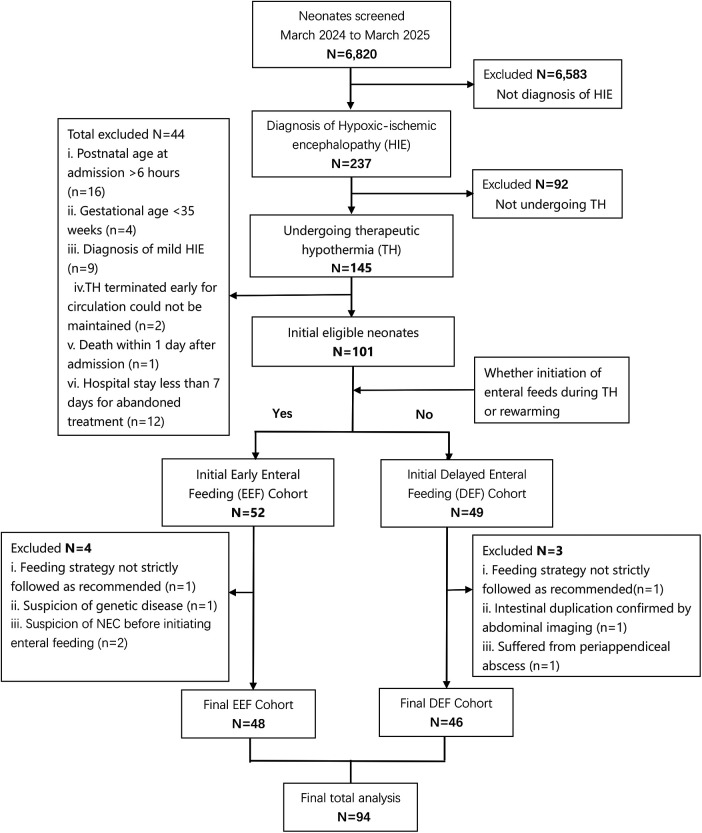
Patient selection flowchart.

A total of 101 neonates were evaluated during the study period, and 7 were excluded (2 failed to strictly follow the standardized feeding strategy; 1 was suspected of having a genetic disease, 2 was suspected of developing NEC before the initiation of enteral feeding, 1 was subsequently considered to have intestinal duplication malformation, and 1 was considered to have periappendiceal abscess, all of which may affect enteral feeding). Finally, 94 neonates were enrolled, including 48 in the early enteral feeding (EEF) group and 46 in the delayed enteral feeding (DEF) group. Only 1 neonate died before the initiation of enteral feeding, and this case was excluded from the outcome analysis (Figure 1).

The demographic characteristics, maternal-related factors, and baseline HIE severity were comparable between the two groups (all *P* > 0.05) (Table 1). The mean gestational age was 39.1 ± 1.4 weeks in the EEF group and 38.7 ± 1.3 weeks in the DEF group; the mean birth weight was 3.1 ± 0.4 kg in the EEF group and 3.1 ± 0.4 kg in the DEF group; the median 5-minute Apgar score was 8.8 ± 1.7 vs. 8.7 ± 1.6 in two groups (all *P* > 0.05).

**Table 1:** Baseline characteristics between the two cohorts, [*n* (%), *x¯* ± *s*, *M* (IQR)].

Variables	EEF cohort (*n* = 48)	DEF cohort (*n* = 46)	*P*-value
Infant factors			
Male sex	23 (47.9)	26 (56.5)	0.404
Gestational age (weeks)	39.1 ± 1.4	38.7 ± 1.3	0.145
Birth weight (kg)	3.1 ± 0.4	3.1 ± 0.4	0.988
Age at admission (hours)	2.0 (1.0, 3.0)	2.0 (2.0, 3.0)	0.874
Apgar score			
At 1 min	7.2 ± 2.2	7.1 ± 2.2	0.788
At 5 min	8.8 ± 1.7	8.7 ± 1.6	0.784
At 10 min	9.5 ± 0.9	9.3 ± 1.0	0.518
First postnatal blood gas within 1 h of birth (or cord blood when available)^a^			
pH	7.1 ± 0.1^b^	7.1 ± 0.2^b^	0.323
Base deficit (mmol/L)	12.7 ± 3.2^b^	12.3 ± 4.4^b^	0.671
Lactate (mmol/L)	8.5 ± 2.3^b^	7.5 ± 2.3^b^	0.095
Neonatal blood gas at admission			
PH	7.3 ± 0.1	7.3 ± 0.1	0.193
Base deficit (mmol/L)	7.5 ± 3.4	7.3 ± 4.9	0.803
Lactate (mmol/L)	4.2 ± 2.8	5.6 ± 4.2	0.063
Sarnat clinical stages of HIE			
Moderate HIE	41 (85.4)	42 (91.3)	0.375
Severe HIE	7 (14.6)	4 (8.7)	
Maternal factors			
Abnormal amniotic fluid	26 (54.2)	18 (39.1)	0.144
Premature rupture of membranes	10 (20.8)	15 (32.6)	0.196
Placental abnormalities	1 (2.1)	5 (10.9)	0.107*
Umbilical cord abnormalities	3 (6.3)	3 (6.5)	>0.999*
Intrauterine distress	25 (52.1)	21 (45.7)	0.533
Maternal gestational diabetes	12 (25.0)	12 (26.1)	0.904

Base deficit values are presented as positive numbers, consistent with the terminology used in the Methods section. Abnormal amniotic fluid: Includes meconium-stained amniotic fluid (Grade I/II/III) and blood-tinged amniotic fluid; Umbilical cord abnormalities: Encompasses hypercoiled cord, cord prolapse, exposed umbilical vessels, and marked cord edema; Placental abnormalities: Defined as placental abruption, placental calcification, placenta previa, or velamentous cord insertion.

a Umbilical cord blood gas data were available for a subset of infants (46 in EEF group, 38 in DEF group) due to the outborn nature of the cohort. For infants without cord blood gases, the first postnatal blood gas within 1 h of birth was used.

b Mean values were calculated from infants with measurable values. The following extreme values were excluded from mean calculations: pH and base deficit below detectable limit (3 in EEF group, 1 in DEF group); lactate above detectable limit (1 in each group). These excluded cases reflect the most severely affected infants and are not captured in the reported means.

* Fisher’ exact test.

There were significant differences in auxiliary treatment indicators reflecting disease severity between the two groups: the DEF group had a significantly higher proportion of neonates receiving invasive mechanical ventilation (28.3% vs. 6.3%), hemodynamic support (82.6% vs. 29.2%), and PICC placement (50.0% vs. 14.6%) (all *P* < 0.001). These imbalances are not a limitation of study design but rather provide direct quantitative evidence of the indication confounding phenomenon—i.e., “delayed feeding in more severely ill neonates.” Among the 94 enrolled infants, only one (1.1%) in the DEF group did not receive antibiotics during hospitalization; all remaining infants received prophylactic or therapeutic antibiotics as clinically indicated. No infants received human milk at feeding initiation. At discharge, human milk feeding rates remained low (EEF: 52.1%, DEF: 41.3%), reflecting ongoing challenges with milk access in this population. In addition, the time to initiate feeding was significantly earlier in the EEF group than in the DEF group [2.0 (1.0–2.0) days vs. 5.0 (4.0–5.0) days, *P* < 0.001] (Table 2).

**Table 2:** Adjunctive therapies and feeding practices between the two cohorts, [*n* (%), *x¯* ± *s*, *M* (IQR)].

Variables	EEF cohort (*n* = 48)	DEF cohort (*n* = 46)	*P*-value
Adjunctive therapies			
Mechanical ventilation			
Non-invasive	13 (27.1)	18 (39.1)	0.214
Invasive	3 (6.3)	13 (28.3)	0.005
Duration of antibiotic therapy (days)^a^	6.5 (5.0,10.8)	8.5 (6.0,14.0)	0.220
Duration of PICC (days)	12.0 (11.0,15.0)	9.5 (7.3,13.0)	0.069
Receiving PICC therapy	7 (14.6)	23 (50.0)	<0.001
Hemodynamic support	14 (29.2)	38 (82.6)	<0.001
Feeding practices			
Initial time of first feeding after admission (days)	2.0 (1.0, 2.0)	5.0 (4.0, 5.0)	<0.001
Feeding type			
At initial feeding time			
Human milk	0 (0.0)	0 (0.0)	>0.999*
Formula	48 (100.0)	46 (100.0)	
At discharge feeding time			
Human milk	25 (52.1)	19 (41.3)	0.295
Formula	23 (47.9)	27 (58.7)	
Discharge feeding volume (mL/kg. day)	153.8 ± 19.2	147.7 ± 19.9	0.136

PICC, peripherally inserted central catheters.

a Among the 94 enrolled infants, only one (1.1%) in the DEF group did not receive antibiotics during hospitalization.

* Fisher’ exact test.

### Primary outcome

There was no significant difference in the total incidence of gastrointestinal adverse events (GIAEs) between the two groups (22.9% vs. 21.7%, *P* = 0.891). Similarly, no statistically significant differences were observed in the incidences of feeding intolerance (18.8% vs. 17.4%, *P* = 0.864) and necrotizing enterocolitis (NEC) of stage Ⅱ or higher (4.2% vs. 4.3%, *P* > 0.999) between the two groups (Table 3).

**Table 3:** Primary and secondary outcomes between the two cohorts, [*n* (%), *x¯* ± *s*, *M* (IQR)].

Variables	EEF cohort (*n* = 48)	DEF cohort (*n* = 46)	*P*-value
GIAEs	11 (22.9)	10 (21.7)	0.891
FI	9 (18.8)	8 (17.4)	0.864
NEC	2 (4.2)	2 (4.3)	>0.999*
Late-onset sepsis	0 (0.0)	2 (4.3)	0.144*
Hypoglycemia	3 (6.3)	4 (8.7)	0.711*
Severe neurological injury at discharge	4 (8.3)	4 (8.7)	>0.999*
Hospital weight velocity (g/day)	16.1 ± 10.4	12.5 ± 9.9	0.086
Weight at discharge (kg)	3.3 ± 0.4	3.3 ± 0.4	0.689
Days to full enteral feeding (days)	9.0 (8.0, 11.0)	11.0 (9.8, 14.0)	0.011
Parenteral nutrition duration (days)	10.0 (8.0, 11.8)	12.0 (10.0, 14.3)	0.004
Length of hospital stay (days)	14.0 (10.0, 17.0)	15.0 (12.0, 21.0)	0.217
Total costs (USD)	3,637 ± 1,248	4,672 ± 1,503	<0.001

GIAEs, gastrointestinal adverse events; FI, feeding intolerance; NEC, necrotizing enterocolitis.

* Fisher’ exact test.

The time to achieve full enteral feeding [9.0 (8.0–11.0) days vs. 11.0 (9.8–14.0) days, *P* = 0.011] and the duration of parenteral nutrition [10.0 (8.0–11.8) days vs. 12.0 (10.0–14.3) days, *P* = 0.004] were significantly shorter in the EEF group than in the DEF group. There were no significant differences between the two groups in the incidences of late-onset sepsis, neonatal hypoglycemia, and severe neurological injury, as well as in hospital stay, weight gain rate, and discharge weight (all *P* > 0.05). Health economic indicators: The total hospitalization cost was significantly lower in the EEF group than in the DEF group (3,637 ± 1,248 USD vs. 4,672 ± 1,503 USD, *P* < 0.001) (Table 3).

**Figure 2:**
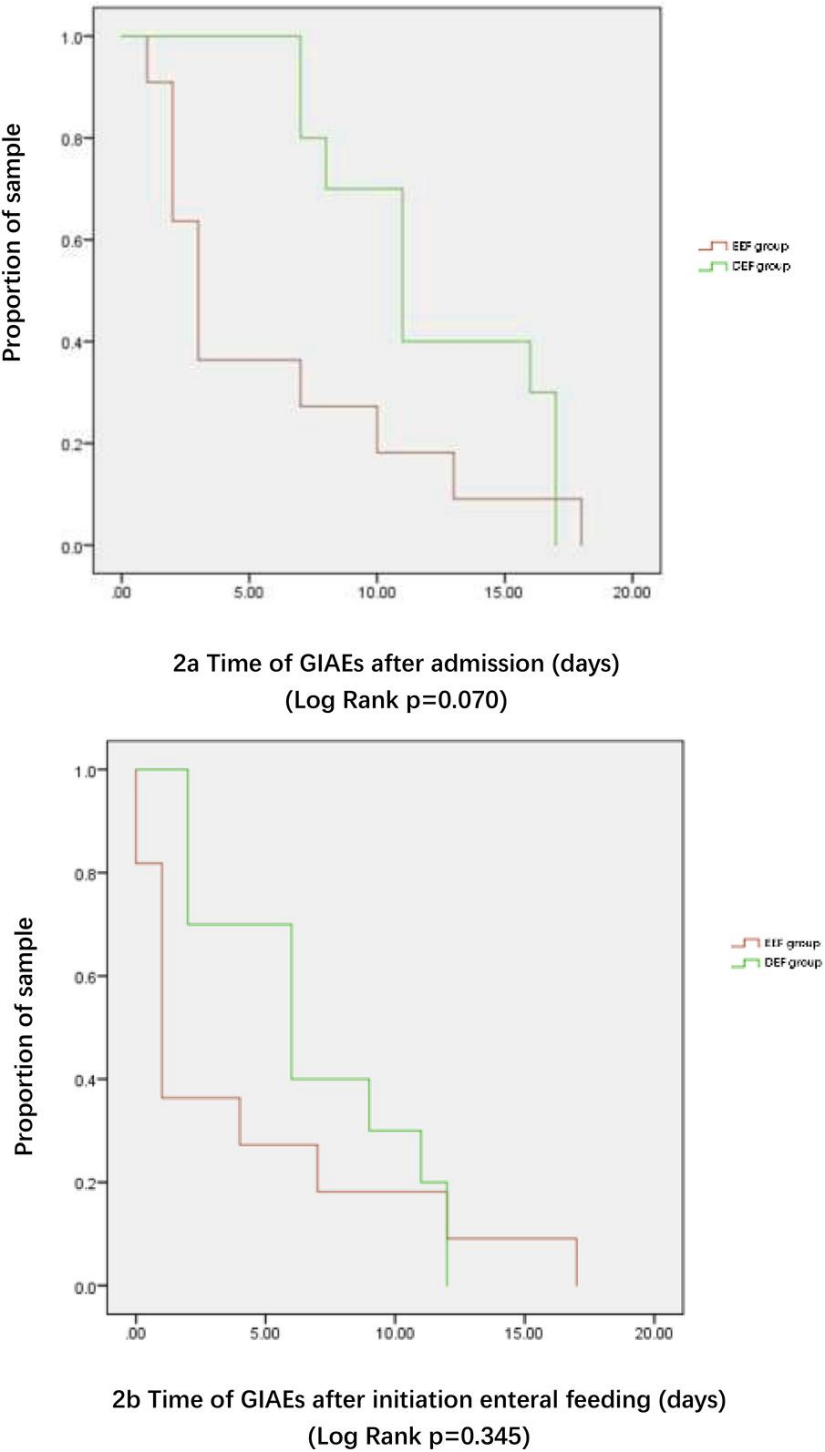
Kaplan-Meier curves showing **(A)** time of GIAEs after admission and **(B)** time of GIAEs after initiation of enteral feeding.

Time-to-event analysis indicated that GIAEs occurred earlier in the EEF cohort relative to both times of admission (median 3.0 vs. 11.0 days) and feeding initiation (median 1.0 vs. 6.0 days), although these differences were not statistically significant (log-rank *P* = 0.070 and *P* = 0.345, respectively; Figure 2).

### Subgroup analyses

After stratification by hypoxic-ischemic encephalopathy (HIE) severity, ventilation mode, and hemodynamic status, there was no significant difference in the incidence of gastrointestinal adverse events (GIAEs) between the two groups within each subgroup (all *P* > 0.05) (Table 4). For descriptive purposes only, the severe HIE subgroup included 11 infants (7 in the EEF group and 4 in the DEF group). The overall incidence of NEC in this subgroup was 18.2% (2/11). Due to the extremely small sample size (*n* = 11), no inferential statistics were performed, and no conclusions regarding feeding safety in this subgroup can be drawn. This analysis is presented solely to highlight the high-risk nature of this population, not to compare feeding strategies.

**Table 4:** Subgroup analyses of adverse events according to disease severity and adjunctive therapies, [*n* (%)].

Variables	EEF cohort *n*/*N* (%)	DEF cohort *n*/*N* (%)	*P*-value
Disease severity			
Severe HIE			
GIAEs	2/7 (28.6)	1/4 (25.0)	0.999*
FI	1/7 (14.3)	0/4 (0.0)	0.864*
NEC	1/7 (14.3)	1/4 (25.0)	>0.999*
Moderate HIE			
GIAEs	9/41 (22.0)	9/42 (21.4)	0.954*
FI	8/41 (19.5)	8/42 (19.0)	0.957*
NEC	1/41 (2.4)	1/42 (2.4)	>0.999*
Mechanical ventilation			
Invasive MV			
GIAEs	1/3 (33.3)	2/13 (15.4)	0.489*
FI	1/3 (33.3)	1/13 (7.7)	0.350*
NEC	0/3 (0.0)	1/13 (7.7)	>0.999*
Non-invasive MV			
GIAEs	1/13 (7.7)	2/18 (11.1)	>0.999*
FI	0/13 (0.0)	1/18 (5.6)	>0.999*
NEC	1/13 (7.7)	1/18 (5.6)	>0.999*
Hemodynamic support			
Requiring			
GIAEs	4/14 (28.6)	9/38 (23.7)	0.729*
FI	3/14 (21.4)	8/38 (21.1)	>0.999*
NEC	1/14 (7.1)	1/38 (2.6)	0.470*
Non-requiring			
GIAEs	7/34 (20.6)	1/8 (12.5)	>0.999*
FI	6/34 (17.6)	0/8 (0.0)	0.576*
NEC	1/34 (2.9)	1/8 (12.5)	0.348*

GIAEs, gastrointestinal adverse events; FI, feeding intolerance; NEC, necrotizing enterocolitis; MV, mechanical ventilation.

* Fisher’ exact test.

### Multivariate logistic regression analysis

The Hosmer-Lemeshow test indicated that the multivariate logistic regression model had a good fit (*χ*^2^ = 4.12, *P* = 0.723), suggesting high reliability of the model (Table 5). After adjusting for potential confounding factors including HIE severity, invasive mechanical ventilation, and hemodynamic support, early enteral feeding was not independently associated with GIAEs [odds ratio [OR] = 0.75, 95% confidence interval (95% CI): 0.23–2.44, *P* = 0.636]. In addition, no significant statistical associations were observed between severe HIE, invasive mechanical ventilation, hemodynamic support, and GIAEs (all *P* > 0.05). Notably, the 95% confidence intervals (95% CI) for all variables were relatively wide, which reflects the impact of insufficient statistical power in this study.

**Table 5:** Multivariate analysis of potential factors related to GIAEs.

Variables	*β*	S.E.	Wald	*P*-value	OR	95% CI
Early enteral feed	−0.283	0.599	0.224	0.636	0.753	0.233–2.436
Severe HIE	0.274	0.724	0.143	0.705	1.315	0.318–5.434
Invasive MV	−0.300	0.738	0.166	0.684	0.741	0.175–3.144
Hemodynamic support	−0.552	0.599	0.849	0.357	0.576	0.178–1.863
Constant	−0.843	1.053	0.641	0.423	0.431	

GIAEs, gastrointestinal adverse events; HIE, hypoxic-ischemic encephalopathy; MV, mechanical ventilation.

## Discussion

This study contributes to the existing literature by quantifying how indication confounding shapes enteral feeding decisions in neonates with HIE undergoing therapeutic hypothermia—a methodological issue that has received limited direct attention in prior observational studies. While previous investigations have generally reported that early feeding appears safe, most have not adequately accounted for the inherent differences in disease severity between infants fed early vs. those fed late (6–9). By directly measuring and adjusting for key severity indicators, our findings provide a more nuanced understanding of how baseline clinical status shapes both feeding decisions and subsequent outcomes.

A central finding is that infants in the delayed feeding group were markedly sicker at baseline, as reflected by significantly higher rates of invasive mechanical ventilation, hemodynamic support, and PICC placement (all *P* < 0.001). This pattern confirms what clinicians intuitively recognize—that feeding is withheld or postponed in the most vulnerable infants—and provides quantitative evidence of the indication bias that has complicated interpretation of earlier work. Previous studies have approached this issue in varying ways but stopped short of fully characterizing or adjusting for these imbalances. A nationwide survey from Poland, for example, qualitatively described reasons for delayed feeding without quantifying their association with clinical indicators (3); an Omani cohort stratified by HIE severity but did not include mechanical ventilation in its multivariable models (8); a South African study collected relevant physiologic data but found no significant between-group differences and did not pursue stratified analyses (9); and the study by Zhong and colleagues from Chongqing did not adjust for endotracheal intubation status (12). By contrast, we systematically collected and adjusted for a range of objective severity indicators through both stratified analyses and multivariable regression, allowing us to estimate the magnitude of confounding more directly.

In terms of clinical outcomes, univariate analysis showed no significant differences between groups in gastrointestinal adverse events, feeding intolerance, or NEC, consistent with several prior reports (5, 6, 12). The higher NEC incidence in our cohort (4.3%) compared with some previous studies (8, 9) may reflect differences in surveillance intensity. Our unit employs an intensive abdominal monitoring protocol for HIE infants, including daily abdominal circumference measurements, gastric residual assessment, and bowel sound auscultation, which may increase sensitivity for detecting early or subtle NEC. It is also possible that the reported NEC incidence in prior studies may be underestimated due to less frequent monitoring or differences in case ascertainment. In our study, all NEC diagnoses were made according to the modified Bell criteria (stage ≥ II) and independently adjudicated by trained evaluators. We therefore believe the higher incidence reflects more complete case detection rather than over-diagnosis.

After multivariable adjustment for HIE grade, ventilatory support, and other confounders, early feeding was not independently associated with gastrointestinal adverse events. This finding highlights the extent to which unadjusted analyses can be misleading and underscores the importance of accounting for disease severity when interpreting observational data in this population.

From a clinical standpoint, early feeding was associated with shorter parenteral nutrition duration and lower total hospitalization costs—findings consistent with several previous studies (6, 10, 12). However, unlike some earlier reports, we did not observe a statistically significant reduction in hospital length of stay. This may reflect limited power, center-specific discharge practices, or the possibility that length of stay is a less sensitive metric than parenteral nutrition duration in this setting.

Subgroup analyses suggested no differences in gastrointestinal adverse events across strata defined by HIE severity, ventilation status, or hemodynamic support. The high NEC rate observed in the severe HIE subgroup (18.2%) should be interpreted with caution, as this analysis included only 11 infants and is exploratory in nature. While a South African study noted a similar pattern, it did not report separate analyses for severe HIE infants (9); a Turkish RCT stratified by Sarnat stage but examined oral motor training rather than feeding timing, precluding direct comparison (10).

Building on the comparative summary in Table 6, we offer several considerations for future research. Well-designed multicenter randomized trials with stratification by HIE severity and respiratory/hemodynamic support status would provide the most definitive evidence by eliminating indication confounding at the design stage. Data collection should systematically capture not only binary indicators of organ support but also their duration and intensity, along with detailed feeding protocols such as initiation timing, initial volume, advancement rate, and milk type. Outcome measurement could be strengthened by incorporating standardized feeding assessment tools and, where feasible, instrumental evaluations such as video-fluoroscopic swallowing studies to improve detection of subtle aspiration; extended follow-up through early childhood would also clarify the long-term implications of early feeding strategies. Finally, transparent reporting of baseline severity indicators and adjusted effect estimates with confidence intervals will help readers assess the potential influence of residual confounding and the precision of findings.

**Table 6:** Comparison of key studies on enteral feeding during therapeutic hypothermia in neonates with HIE.

Study	Design	Sample	Key findings	Confounding control	Main contribution/limitation
Present study (2026)	Retrospective cohort	94	Early feeding: shorter PN, lower costs; no independent association with GIAEs	Adjusted for HIE severity, ventilation, vasoactive agents	Quantified indication confounding; residual confounding possible
Thyagarajan et al. (5) (2015)	Retrospective cohort	85	No NEC; longer stay with early feeding	None	Early feasibility; no confounding control
Chang et al. (6) (2018)	Retrospective case-control	34	Early feeding: shorter PN, stay	None	Supported short-term benefits; no severity adjustment
Zhong et al. (12) (2021)	Retrospective cohort	95	Early feeding: shorter PN, stay; no NEC increase	None (baseline imbalances)	Early Chinese experience; no confounding control
Malviya et al. (8) (2025)	Retrospective cohort	187	Severe HIE: delayed feeding, longer to full feeds; 1 NEC case	HIE severity stratified; MRI score	Large sample; did not adjust for ventilation/vasoactive agents
Ilhaam et al. (9) (2025)	Retrospective cohort	48	Feeding intolerance linked to higher HIE severity, longer stay	HIE severity analyzed	No adjustment for ventilation/vasoactive agents
Bozkaya et al. (10) (2023)	RCT	100	Oral motor intervention: faster full oral feeds in severe HIE	Stratified by Sarnat stage	RCT eliminated indication bias; intervention was oral motor training
Shui et al. (7) (2024)	Retrospective cohort	179	Feeding timing, initial volume, advancement rate predicted FI	Ventilation in model (*P* = 0.062)	Predictive model for FI; HIE severity not in final model
Arman et al. (26) (2025)	RCT	60	Early feeding: earlier full feeds, shorter stay, less FI; no NEC	Balanced baseline; blinded assessment	Physiological evidence (NIRS/Doppler); small sample for rare outcomes
Present study (2026)	Retrospective cohort	94	Early feeding: shorter PN, lower costs; no independent association with GIAEs	Adjusted for HIE severity, ventilation, vasoactive agents	Quantified indication confounding; residual confounding possible

PN, parenteral nutrition; GIAEs, gastrointestinal adverse events; NEC, necrotizing enterocolitis; FI, feeding intolerance; RCT, randomized controlled trial; NIRS, near-infrared spectroscopy.

While confounding by indication is an important consideration in observational studies of feeding timing during therapeutic hypothermia, randomized controlled trials provide complementary evidence that helps elucidate the underlying physiology. In a prospective randomized study by Arman et al. (26), infants with hypoxic-ischemic encephalopathy who received minimal enteral nutrition during therapeutic hypothermia were compared with those who remained unfed. Using near-infrared spectroscopy and Doppler ultrasonography, the authors found that early minimal feeding did not adversely affect cerebral or mesenteric oxygenation, and no infant in either group developed necrotizing enterocolitis. Moreover, the fed group achieved full enteral nutrition significantly earlier and had shorter hospital stays, with a lower incidence of feeding intolerance. These physiological data suggest that early feeding during therapeutic hypothermia is feasible and may confer genuine benefits beyond those attributable to differences in baseline illness severity. Taken together with our observational findings, the evidence supports the safety and potential advantages of early enteral feeding in clinically stable infants with moderate hypoxic-ischemic encephalopathy, while acknowledging that the magnitude of benefit observed in observational studies may be influenced by residual confounding.

The mean Apgar scores and blood gas values in our cohort are higher than those reported in landmark cooling trials such as the NICHD study. Several factors explain this difference. First, as a children's hospital without obstetric services, all infants were outborn, and Apgar scores obtained from referring hospitals may be subject to reporting bias. Second, our institutional protocol adopted a slightly broader acid-base threshold (base deficit ≤12 mEq/L) in combination with clinical encephalopathy, reflecting real-world practice. Consequently, our cohort is enriched with moderate HIE cases (88.3%), whereas severe HIE accounted for only 11.7%. This distribution differs from that of classic randomized trials but aligns with the patient population commonly encountered in everyday clinical practice. Therefore, our findings are most applicable to infants with moderate HIE, and caution should be exercised when extrapolating these results to populations with predominantly severe HIE.

Several limitations should be acknowledged. First, despite multivariable adjustment for key confounders, residual confounding cannot be ruled out. More robust causal inference methods (e.g., propensity score matching) were not feasible given the sample size constraints of this exploratory study. Therefore, our findings should be interpreted as associations rather than causal effects, and are best viewed as hypothesis-generating for future research. This limitation is compounded by the marked baseline imbalances between groups, which further underscore the presence of confounding by indication. Second, excluding infants with hospital stay <7 days (including one early death) introduced survivor bias, as these infants represented the sickest neonates. Their exclusion may have biased safety outcomes in favor of early feeding, potentially overestimating its safety. A formal sensitivity analysis could not be performed due to incomplete data for transferred-out cases. Survivor bias typically skews results toward the intervention received by healthier patients; thus, if our findings show no increased risk despite this bias, the true effect may be even more conservative. Readers should interpret safety outcomes with this in mind, and future prospective studies are needed. Third, feeding timing was treated as a fixed exposure in the main analysis, which may introduce immortal time bias. While a time-dependent analytical approach would be preferable, it was not feasible in this study due to the small sample size and exploratory nature. Future studies with larger cohorts should employ time-dependent methods to more accurately assess the relationship between feeding timing and outcomes. Fourth, with only 21 outcome events, the events-per-variable ratio was approximately 7, which is below the conventional threshold for confirmatory analyses. The wide confidence intervals reflect limited statistical power, and findings should be interpreted as hypothesis-generating. Fifth, extreme blood gas values (pH and base deficit below detectable limits in 4 infants; lactate above detectable limits in 2 infants) were excluded from mean calculations because exact values could not be obtained. This may have led to an underestimation of the true severity of acidosis in the most severely affected infants. Sixth, we did not collect long-term follow-up data on neurodevelopment or feeding tolerance beyond hospital discharge. Seventh, the higher rate of PICC placement in the delayed feeding group likely reflects a combination of greater baseline illness severity (as evidenced by higher rates of invasive ventilation and hemodynamic support) and the nutritional consequences of delayed feeding. Delayed enteral feeding inevitably prolongs exclusive parenteral nutrition, increasing the need for central venous access such as PICC lines. This observation indirectly supports the potential benefit of early feeding in reducing central line dependence and associated risks. We did not formally test whether catheter status modified the relationship between feeding timing and outcomes, which remains a limitation.

In summary, this study provides quantitative evidence that indication confounding is associated with the observed relationship between enteral feeding timing and outcomes in HIE infants undergoing therapeutic hypothermia. While we observed an association between early feeding and certain benefits, the concentration of adverse events in the most severely affected infants underscores the need for individualized, severity-stratified approaches. Future research should prioritize designs that minimize confounding at the outset—such as propensity score methods in large observational datasets or, ideally, randomized controlled trials—and employ standardized, comprehensive data collection to support more definitive conclusions.

## Conclusion

This study quantifies the extent of indication confounding in enteral feeding decisions for neonates with HIE undergoing therapeutic hypothermia, confirming that sicker infants are systematically more likely to receive delayed feeding. After adjusting for disease severity, early feeding was not independently associated with gastrointestinal adverse events, suggesting that previously reported benefits may reflect baseline differences rather than a true protective effect of early feeding.

For clinically stable infants with moderate HIE—those without need for invasive ventilation or hemodynamic support—early initiation of minimal enteral feeds (≤20 mL/kg/day) with gradual advancement appears safe and is associated with shorter parenteral nutrition duration and reduced hospitalization costs. However, given the potential survivor bias introduced by excluding infants with hospital stay <7 days, these findings should be interpreted with caution and require confirmation in prospective studies.

By systematically characterizing indication bias, this study offers a methodological framework for interpreting observational evidence in this population. Future research should employ rigorous designs with adequate sample size, comprehensive severity adjustment, and extended follow-up to better delineate the independent effects of feeding timing on both short-term outcomes and long-term neurodevelopment.

## Data Availability

The raw data supporting the conclusions of this article will be made available by the authors, without undue reservation.
