# Supplementary material for: Enteral feeding timing and clinical outcomes in neonates with hypoxic-ischemic encephalopathy undergoing therapeutic hypothermia: a retrospective cohort study highlighting the challenge of confounding by indication
Source: Front Pediatr. 2026 Apr 23;14:1818709. doi: 10.3389/fped.2026.1818709 (PMC13149273; doi:10.3389/fped.2026.1818709)
Supplement: Supplementary file 1 [file Datasheet1.pdf]

Recommended enteral feeding protocol between the two cohorts.

Content	EEF Cohort	DEF Cohort
Initiation time of enteral feeding	Feed if medically feasible during TH and rewarming	Feed if medically feasible after TH
Feeding delivery method	Oral feeding intermittent feeding every 3 hours (20~30min) or nasogastric tubes feeding bolus every 3 hours (30 minutes-1 hour) if infants unable to start oral feeds after evaluation of ability for sucking and swallowing daily	Same
Feeding type of milk at initiation [a]	Breast milk or common formula [b] if mothers` milk cannot be available	Same
Volume of initiation enteral feeding	10~20 ml/kg/d	Same
Speed of feeding advancement [a]	(1) 10~20 ml/kg/d after starting feeds for 3 days and the maximum speed is 30 ml/kg/d (first choice)<br>(2) 5~10 ml/kg/d for 3 days and the maximum speed is 20 ml/kg/d (second choice) when infants developed FI	Same
Frequency of feeding advancement a	(1) Advancement of milk volume each 1 day up to the goal of full enteral feeds (first choice)<br>(2) A short fast if necessary, then advancement of milk volume each 1~2 days up to the goal of full enteral feeds (second choice) when infants developed FI	Same
Goal of full enteral feeds	120 ml/kg/d	Same

a All second choice should be only given to infants when clinicians consider them may occur feeding intolerance who cannot continue first choice.

b Common formula is defined as formula for term and near term infants (GA⩾35 weeks) containing energy as 67~68 kcal per each 100 ml.
